# Effects of PRP injection combined with intersecting femoral head decompression versus combined with multiple core decompression in the treatment of avascular necrosis of femoral head—a single-center retrospective cohort study

**DOI:** 10.1186/s12891-025-08679-9

**Published:** 2025-05-02

**Authors:** Lei Sun, Ziyao Xu, Zhongyuan Zhang, Jinwei Zhao, Mengshuai Sun, Guangling Gao, Fang Gao, Hongjiang Jiang, Changjun Ju

**Affiliations:** Department of Orthopaedics, Wendeng Orthopaedic Hospital, Weihai, Shandong Province 264400 China

**Keywords:** ANFH, Intersecting femoral head decompression, Core decompression, PRP

## Abstract

**Background:**

Avascular necrosis of femoral head (ANFH) is a localized destruction of the femoral head caused by a variety of complex reasons, which eventually progresses to collapse of the femoral head and loss of joint function. Platelet-rich plasma(PRP), an autologous platelet concentrate rich in growth factors, has been widely used in the treatment of ANFH because of its ability to promote osteogenesis. PRP injection is often combined with multiple surgical approaches to treat ANFH. The objective of this study is to evaluate the effect of PRP injection combined with intersecting femoral head decompression versus combined with multiple core decompression (MCD) in the treatment of ANFH.

**Methods:**

A retrospective cohort analysis comparing two surgical interventions was conducted between January 2020 and December 2020. Specifically, 25 patients with ARCO stage II non-traumatic ANFH received PRP injection combined with intersecting femoral head decompression, while another 25 patients underwent PRP injection plus MCD. Postoperative functional outcomes were assessed using the Harris Hip Score (HHS) and Visual Analogue Scale (VAS), while radiographic progression was evaluated through standardized X-ray and computed tomography (CT) imaging protocols. Functional and radiographic results were compared between the two groups.

**Results:**

All patients were followed up without occurrence of complications such as infection and thrombosis. The mean follow-up time was 29.7 ± 3.4 months. HHS of patients treated by intersecting femoral head decompression at 12 months and 24 months postoperatively (78.7 ± 4.1 and 79.8 ± 5.0) were significantly higher than those of patients treated by MCD (75.9 ± 5.4 and 76.6 ± 5.1) (*P* < 0.05). VAS of patients treated by intersecting femoral head decompression at 12 months and 24 months postoperatively (2.7 ± 0.8 and 2.6 ± 0.8) were significantly lower than those of patients treated by MCD (3.3 ± 0.8 and 3.1 ± 0.8) (*P* < 0.05).. HHS and VAS were improved postoperatively in both groups (*P* < 0.05). According to radiographic changes, the proportion of patients with aggravation of necrosis in patients treated by intersecting femoral head decompression (12%) was lower than that (24%) in patients treated by MCD at 24 months postoperatively (*P* > 0.05), but the difference is not significant.

**Conclusion:**

Unlike conventional core decompression, PRP can penetrate slowly into the cancellous bone of the femoral head through drilled pores in patients treated by intersecting femoral head decompression, thus extending duration of action of PRP. Compared with PRP injection combined with MCD, PRP injection combined with intersecting femoral head decompression had better functional and radiographic outcomes in our study.

## Background

ANFH is a prevalent disease for orthopedics that leads to total hip arthroplasty (THA) in 80% of cases [1]. The interruption of the blood supply and increased intraosseous pressure lead to cellular death in trabecular bone and marrow, then cause collapse of articular cartilage and subsequently secondary osteoarthritis [2]. The course of ANFH is rarely reversible, eventually leading to loss of joint function and THA [3]. Despite satisfactory results of THA, 13.8% of patients received revision surgery [4], therefore enough attention should be paid to slowing down the progression of the disease for the early stage of ANFH. Without treatment, impairment of joint function and pain may happen within 2 years [5]. Due to insufficient understanding of the pathogenesis and etiology of ANFH, many methods were used such as the administration of bisphosphonates, vasodilators, bone mesenchymal stem cells (BMSCs), PRP and hip-preserving surgery.

Core decompression was the most common hip-preserving surgery whose purpose was to reduce the intraosseous pressure and improve the blood supply of the femoral head. Procedures include traditional single core decompression and MCD. Though Liu reported there was no statistical difference in the outcome of radiographic progression by various core decompression techniques [6], the incidence of complications is not the same. Li thought the location or size of the drill hole may directly affect whether a fracture occurs after surgery [7]. Traditional single core decompression is performed using an 8–10 mm ring drill to reach the necrotic area of the femoral head from the cortex below the greater trochanter through the femoral neck under X-ray fluoroscopy, thereby removing the necrotic bone tissue. The disadvantage of single core decompression is that there are certain complications, such as damage to articular cartilage or the occurrence of intertrochanter fractures. Surgeons later improved the procedure by replacing the traditional drill with a 3–4 mm diameter Kirschner's pin for multidirectional and multipored decompression. In 2004, Mont first reported a success rate of 80% for the MCD technique in patients with ANFH before articular surface collapse in a 2-year follow-up [8]. Compared with single core decompression, MCD can reach more necrotic areas with more complete decompression and less surgical trauma.

Though core decompression can reduce intraosseous pressure, Brown found that the rate of bone resorption tended to be faster than the formation of new bone in the process of femoral head repair after decompression, which further weakened the mechanical support of the already weak subchondral bone [9]. Therefore, it is more likely to cause femoral head collapse, and the collapse rate is still about 30%. Core decompression alone may not be effective in improving pain and function in the long term, especially for the cases of mid-stages (ARCO stage II/III) [10]. To promote new bone formation after decompression, core decompression can be combined with MSCs, PRP, and growth factors injection. Li reported stem cell therapy combined with core decompression was more effective in preventing collapse than core decompression alone [11].

PRP has been widely used in knee osteoarthritis, delayed healing or non-healing and tendon injuries. Aditya reported combining PRP instillation with core decompression improved functional outcome and delayed progression in early-age ANFH [12]. The multiple growth and differentiation factors released from PRP at the injury site can promote the natural healing process [13]. These factors include platelet-derived growth factor (PDGF), transforming growth factor-b (TGF-b), insulin-like growth factor, vascular endothelial growth factor (VEGF), hepatocyte growth factor, endothelial growth factor, and platelet factor 4 [13].

In procedures of single core decompression, PRP was injected through the bone channel to the lesion area of the femoral head. This approach tends to result in the loss of PRP, because PRP can flow away from the bone channel as the patient's position changes, which reduces local cytokine concentration. In procedures of MCD, PRP was injected into the articular cavity of hip joint, but growth factors need to penetrate through the articular cartilage to take effect, which reduces local cytokine concentration in lesion area too. There is not much literature on penetration efficiency of cytokines in PRP. It also remains unclear whether promoting cytokine penetration through articular cartilage is helpful for tissue repair within the femoral head. Our research hypothesis is that drilling some pores in articular cartilage can accelerate cytokine penetration and thus promote tissue repair. Based on the hypothesis, the authors improved the procedures of MCD. In addition to the three Kirschner pins placed along the femoral neck, we placed another 3 Kirschner pins from the anterior aspect of the femoral neck to the lesion area, making sure that these 3 pins intersect with other 3 pins. We expect PRP in articular cavity can get into the femoral head through the pores drilled by Kirschner pins and slowly infiltrates the cancellous bone within the femoral head, thus raising local cytokine concentration and promoting bone regeneration. The aim of this study is to compare the effects of PRP injection combined with intersecting femoral head decompression versus combined with MCD.

## Methods

### Study design

This retrospective cohort study enrolled patients fulfilling the predefined inclusion/exclusion criteria, whose medical records were systematically reviewed between January 2020 and December 2020 at Wendeng Orthopedic Hospital's Orthopedic Department. The study was approved by the institutional review board, following the ethical standards of the Declaration of Helsinki. All patients submitted written consent prior to inclusion in the study.

### Patient information

In this observational retrospective cohort analysis, eligibility criteria encompassed: (1) Between 25 and 60 years old (2) Radiographically confirmed ANFH at ARCO stage II, (3) Ability to provide follow-up data for ≥ 24 months postoperatively (4) failed to respond to previous physical therapies or drugs. Exclusion criteria strictly eliminated: (1) Patients with advanced stages of ANFH (2) Secondary ANFH due to inflammatory disorders or traumatic etiologies, (3) Coexisting malignancy, immunosuppressive therapy, or life-threatening comorbidities (4) Hip surgery history (5) History of intra-articular hip injections within the three months before surgery. After screening, 50 patients met the eligibility criteria and exclusion criteria, which were divided into 2 groups. Patients of Group A (PRP + MCD; *n* = 25) received PRP after MCD. Patients of Group B (PRP + intersecting femoral head decompression; *n* = 25) received PRP after intersecting femoral head decompression. Baseline demographics, preoperative HHS and VAS scores were systematically documented. Standardized radiographic assessments including anteroposterior and lateral hip radiographs were performed preoperatively.

### Surgical procedures

**Fig. 1:**
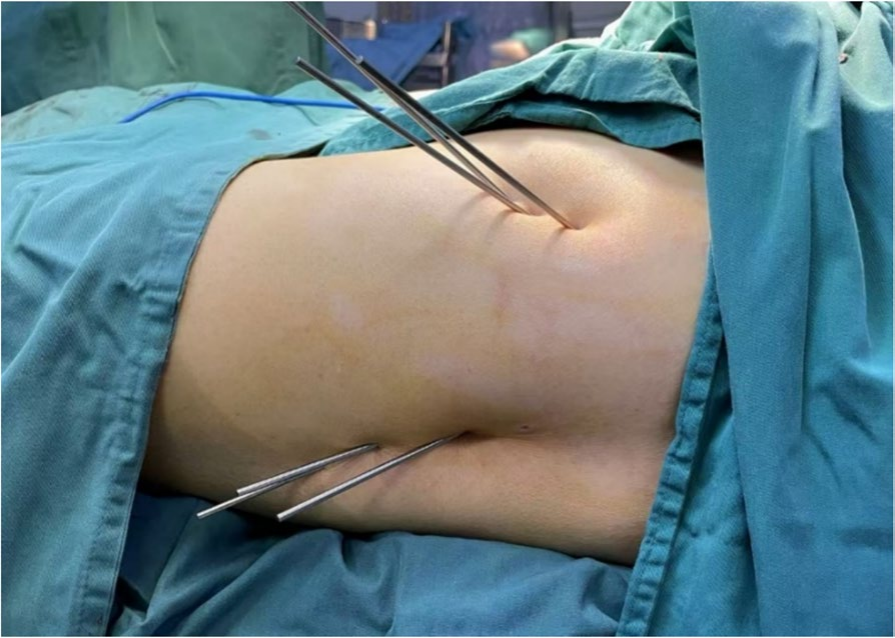
Photograph showed Kirschner pins were inserted from lateral and anterior aspect of hip joint to the lesion area of femoral head

**Fig. 2:**
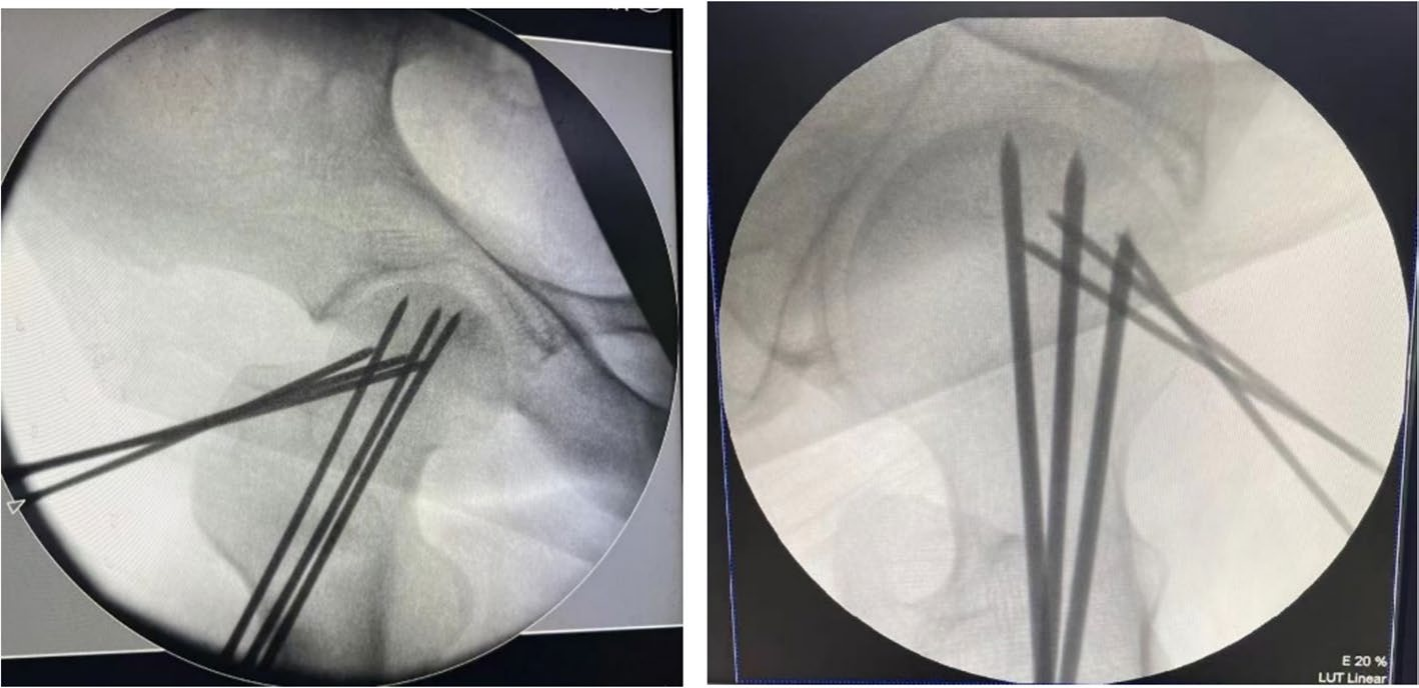
Anteroposterior and lateral X-ray of hip joint showed tips of Kirschner pins reached to the lesion area of femoral head from different directions

All operations were performed by two senior surgeons under spinal anesthesia or general anesthesia. Patients are placed in supine position. In group A, 3 Kirschner pins of 3 mm diameter are inserted from the level of the lesser trochanter to the lesion area of the femoral head along the neck of the femur. The tips of the Kirschner pins reach 3–5 mm below the articular cartilage. In group B, in addition to the 3 Kirschner pins, 3 Kirschner pins of 2.5 mm are inserted from the anterior aspect of the femoral neck to the lesion area. These 3 Kirschner pins should pass through the hip joint capsule, enter the femoral head through the head-neck junction area of the femoral neck and intersect with other 3 pins. The cross angle between pins should be 30–45° as Fig. 1 shows. X-ray results are shown in Fig. 2. After core decompression, PRP injection was operated under X-ray fluoroscopy to make sure PRP was injected into articular cavity.

50 mL of the patient's blood was taken and centrifuged for 15 min at 2000 rpm on a table-top centrifuge (Wego Co, Weihai, China) and 8 mL of PRP was used in articular injection. A total of 3 courses of PRP treatment are needed, with each one being administered 1 week apart.

Patients use crutches after surgery. The affected limb should be kept free from weight-bearing for 3 months, followed by partial weight-bearing for 3 months. Based on radiological results and subjective experience, affected limbs are allowed for weight bearing gradually.

### Radiographic assessment

Based on imaging evaluation, treatment outcomes were categorized as improvement, stabilization, and deterioration. Improvement: the femoral head is morphologically stable, the cystic area is reduced or disappeared; the sclerotic zone is blurred or disappeared, and no osteoarthritis occurs. Stabilization: the femoral head is morphologically stable or collapses less than 2 mm, the cystic area is reduced in size or disappears; the sclerotic zone is blurred or disappears; or mild osteoarthritis is present. Deterioration: collapse of the femoral head > 4 mm, significant osteoarthritis.

### Follow‑up

Preoperative hip function was assessed using the HHS and VAS. Follow-up evaluations were conducted at 12 and 24 months postoperatively, during which clinical outcomes were systematically documented through repeated HHS and VAS assessments. Pelvic radiography and computed tomography (CT) imaging of the affected hip were performed at each follow-up interval to evaluate disease progression and treatment response.

### Statistical analysis

Statistical analyses were performed using IBM SPSS Statistics version 22.0. Quantitative data are presented as mean ± standard deviation (SD). For normally distributed data, means were compared using Student t test between 2 groups. Analysis of variance was applied for more than 2 groups. Mann–Whitney test was used for non-normally distributed data. Categorical variables were expressed by frequency N (%)). The χ2 test or Fisher’s exact probability method was used for group comparisons according to the sample size and expected number in each cell. A *P*-value less than 0.05 was considered statistically significant.

## Results

### General information

A retrospective cohort of 61 patients with ARCO stage II ANFH undergoing core decompression was screened. After exclusion of 11 subjects (4 with advanced stages of ANFH, 4 with secondary ANFH due to trauma, 1 with preoperative malignancy, and 2 lost to follow-up), 50 eligible participants (33 males, 17 females; mean age 40.5 ± 7.4 years) were ultimately included. The etiological distribution was: alcohol-related (21 cases), steroid-induced (15 cases), and idiopathic (14 cases). The median follow-up duration was 29.7 ± 3.4 months. During surveillance, no thromboembolic or postoperative infections occurred, and 4 patients underwent THA due to disease progression. Hip pain while walking is the main cause of poor hip function after surgery (Table 1).

**Table 1:** Patient demographics and clinical characteristics

Characteristics	Group A (*n* = 25)	Group B (*n* = 25)	*p*-value
Age(x ± s, years)	40.9 ± 7.8	40.1 ± 7.2	0.720
Gender (M/F)	17/8	16/9	0.765
BMI (kg/m^2^)	24.3 ± 3.2	24.6 ± 3.8	0.744

Based on Table 1, there was no significant difference in the age, gender, BMI, between two groups (*P* > 0.05, t-test)

### Comparison of HHS between Group A and Group B Table 2

**Table 2:** HHS

	Group A (*n* = 25)	Group B (*n* = 25)	*p*-value
HHS (before surgery)	70.7 ± 4.5	69.1 ± 5.1	0.233
HHS (12 months after surgery)	75.9 ± 5.4	78.7 ± 4.1	0.042
HHS (24 months after surgery)	76.6 ± 5.1	79.8 ± 5.0	0.028
*p*-value	0.017	0.022	

Based on Table 2, there was no significant difference in the preoperative HHS between two groups (*P* > 0.05, t-test). HHS of Group A at 12 and 24 months after surgery is significantly lower than that in Group B(*P* < 0.05, t-test). According to. repeated measures ANOVA, HHS improved after surgery in both groups. (*P* < 0.05)

### Comparison of VAS between Group A and Group B Table 3

**Table 3:** VAS

	Group A (*n* = 25)	Group B (*n* = 25)	*p*-value
VAS (before surgery)	5.0 ± 0.5	4.8 ± 0.4	0.155
VAS (12 months after surgery)	3.3 ± 0.8	2.7 ± 0.8	0.011
VAS (24 months after surgery)	3.1 ± 0.8	2.6 ± 0.8	0.043
*p*-value	0.031	0.011	

Based on Table 3, there was no significant difference in the preoperative VAS between two groups (*P* > 0.05, t-test). VAS of Group A at 12 and 24 months after surgery is significantly higher than that in Group B(*P* < 0.05, t-test). According to. repeated measures ANOVA, VAS decreased after surgery in both groups. (*P* < 0.05,)

### Comparison of radiographic change between Group A and Group B Table 4

**Table 4:** Radiographic change at 24 months postoperatively

	Group A (*n* = 25)	Group B (*n* = 25)	*p*-value
improvement or stabilization	19 (76%)	22 (88%)	
deterioration	6 (24%)	3 (12%)	0.232

Based on Table 4, the proportion of patients with deterioration of radiographic change in Group A (24%) is higher than that (12%) in Group B. but it is not significant. (*P* > 0.05, Fisher’s exact probability method). Insignificance may be caused by insufficient follow-up time or small sample size

**Fig. 3:**
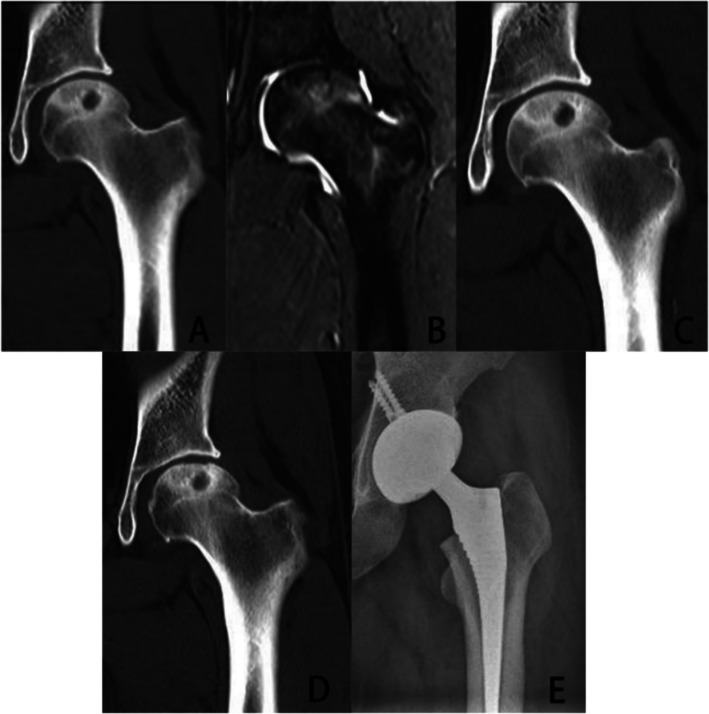
**A**, **B** Preoperative CT and MRI showed ANFH of this patient in ARCO II stage. Localized cystic degeneration of the femoral head can be seen. **C** CT showed there was no obvious progression at 12 months after the patient received PRP + MCD. **D** At 24 months after the patient received PRP + MCD. CT showed collapse of the subchondral bone of the femoral head. **E** Due to osteoarthritis secondary to ANFH, the patient received THA eventually

**Fig. 4:**
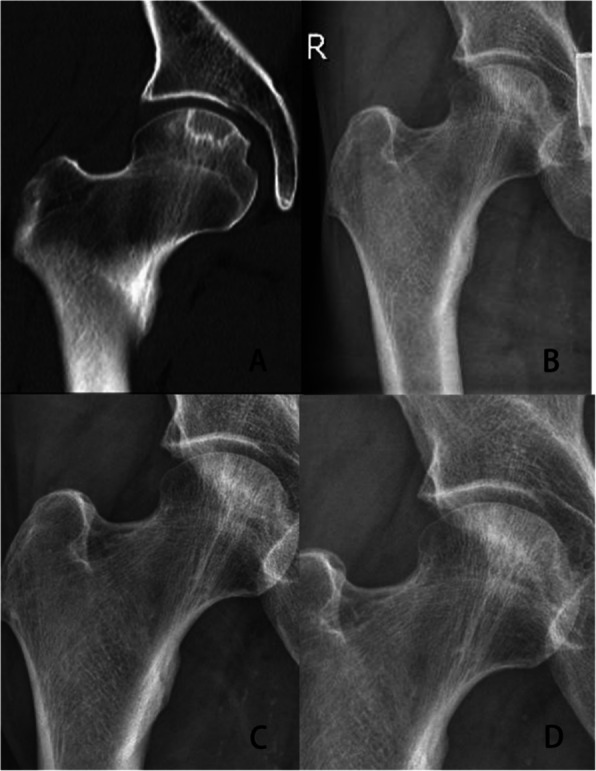
**A**, **B** Preoperative CT and X-ray showed ANFH of this patient in ARCO II stage. Localized cystic degeneration of the femoral head can be seen. **C** X-ray showed there was no obvious progression at 12 months after the patient received PRP + intersecting core decompression. **D** X-ray showed there was no obvious progression at 24 months after the patient received PRP + intersecting core decompression. No collapse of the subchondral bone can be seen

Figures 3 and 4 show the typical radiographic changes of the patients.

## Discussion

Core decompression has been widely used in early stage of ANFH to slow the progression of the disease until now. Core decompression can increase blood flow in the lesion area and reduce intraosseous pressure, thereby reducing pain [14]. Traditional core decompression is single large diameter femoral head decompression, using an 8–10 mm ring drill to reach the necrotic area. Traditional core decompression requires an access hole in the lateral cortex of the femur, which may lead to the occurrence of subtrochanteric fractures. Hua K reported the risk of subtrochanteric fracture is approximately 0.9% [15]. Compared with smaller duct sizes, a duct diameter of 8 mm has been shown to weaken the bone [16]. What’s more, traditional core decompression may lead to impairment to articular cartilage. To overcome these disadvantages, ring drill was replaced by Kirschner's pin with a diameter of 3–4 mm for multidirectional and multipored decompression. Philip reported multiple small diameter drillings increase femoral neck stability compared with single large diameter femoral head decompression technique [9], thus increasing safety. What’s more, larger lesion area can be decompressed by MCD, thus increasing effectiveness. Yang reported MCD can significantly reduce the lesion area of alcoholic femoral head necrosis and idiopathic femoral head necrosis [17].

Core decompression alone may not be effective in improving pain and function in the long term because the rate of bone resorption tends to be faster than the formation of new bone in the process of femoral head repair after decompression. Zhu reported the combination of core decompression and bone grafting with PRP is superior to the approach without PRP [18]. Mechanisms of PRP taking effect in the treatment of ANFH are as following:

1. Promoting osteogenesis and angiogenesis

The essence of the pathogenesis of ANFH is the lack of osteogenic progenitor cells and blood vessels in the necrotic area, so promoting osteogenesis, angiogenesis and reconstructing the bone structure in the necrotic area is the key to the early treatment of ANFH. PRP contains several growth factors that promote osteoinduction and tissue healing such as platelet-derived growth factor (PDGF), transforming growth factor (TGF-β1), transforming growth factor (TGF-β2), vascular endothelial growth factor (VEGF), endothelial growth factor (EGF), insulinlike growth factor (IGF) [19]. Karakaplan reported in a trial of PRP treatment of rabbits with hormonal ANFH, the rate of new bone formation was higher in the PRP-treated group than that in the group treated with bone marrow injection alone and with core decompression alone [20]. VEGF, PDGF and TGF-β that PRP contains are important signaling molecules in the angiogenic pathway. A study reported single injection of PRP accelerated surgical angiogenesis in vascularized necrotic bone [21].

2) Suppression of the inflammatory response

ANFH is an autoimmune disease characterized by chronic inflammation of the joints.

Inflammatory cytokines that promote degradation of mediator were released, followed by autoimmune cells entering the synovium, leading to joint damage [22]. In the experiment of PRP treatment of ANFH mice, the mRNA level and concentration of interleukin 17A (IL-17A), interleukin-1β(IL-1β) and tumor necrosis factor-α (TNF-α) in the synovial cells of mice in the PRP treatment group were significantly reduced, suggesting that PRP treatment inhibits the inflammatory response and reduces the tissue loss and damage by effectively suppressing the expression of the mentioned inflammatory cytokines above in ANFH mice [22].

3) Inhibition of glucocorticoid-induced apoptosis

Overuse of glucocorticoid is considered one of the most common causes of ANFH [23]. ANFH is associated with glucocorticoid-endoplasmic reticulum stress mediated apoptosis in osteoblasts and vascular endothelial cells [24]. Some studies have reported PRP derived exosomes inhibit apoptosis induced by glucocorticoid-endoplasmic reticulum stress in vitro and in vivo. Huang found super active platelet lysate (sPL) which was derived from PRP prevented glucocorticoid-associated femoral head necrosis by regulating autophagy [25].

4) Promoting cartilage repair

PRP has the ability to improve the repair of osteochondral injury, and its repair mechanism is mainly due to a variety of growth factors of high concentrations, which promote the proliferation of osteochondrocytes and secretion of cartilage matrix, thus inducing cartilage regeneration and accelerating the formation of cartilage [26]. PRP can stimulate the migration, proliferation, and differentiation of MSCs into articular chondrocytes, regulate the repair and regeneration of damaged articular cartilage, and promote the lubrication of articular cartilage [27].

5) Analgesic

Aggarwal reported PRP can relieve pain by removing harmful inflammatory factors and interrupting the inflammatory damage process [12].

PRP takes effect in the treatment of ANFH through the mechanisms described above.

PRP may be a non-surgical treatment option for ANFH. Ibrahim reported the patient experienced pain relief and significant functional improvement after treatment of PRP. MRI imaging demonstrated significant improvement in the anatomical structure of the affected femoral head [28]. More often, PRP is combined with core decompression. In a randomized controlled trial reported by Aggarwal [12], 40 patients with ANFH were randomly divided into 2 groups and then treated with core decompression combined with PRP injection or core decompression alone. HHS of patients were significantly higher in the PRP group than in the control group at the final follow-up visit, and the proportion of patients with progression of ANFH in the PRP group was also lower. In our hospital, PRP was used after single large diameter femoral head decompression at an earlier stage., but there are two obvious drawbacks to this approach. Firstly, there is risk of subtrochanteric fracture. Secondly, PRP is injected through the tunnel drilled by 8–10 mm ring drill to the necrotic area. PRP may move through the large-diameter tunnel as the patient's position changes, causing a decrease in PRP concentration in necrotic area. Later multiple small diameter drillings were used in more clinical applications, which reduced damage to bone tissue and decompressed larger lesion area. After MCD, PRP was injected into hip joint. Although many patients had satisfying results in the short term, there was no significant delay in progression of ANFH. The reason may be that the cytokines in PRP inhibit the inflammatory response in the synovium thus relieving the pain, but it’s hard for cytokines to penetrate through the articular cartilage to the lesion area. Low concentration of cytokines in necrotic area may not promote obvious osteogenesis and angiogenesis, resulting in collapse of femoral head in the long term.

Intersecting femoral head decompression is an improvement of MCD, which decompresses larger lesion area and provides a channel for cytokines to enter lesion area. Articular fluid circulates between the articular cavity and the necrotic area of the femoral head, making PRP flow into the necrotic zone continuously to promote osteogenesis and angiogenesis. Just like Fig. 4 shows, there was no obvious progression at 24 months after the patient received PRP + intersecting femoral head decompression. While like Fig. 3 shows, at 24 months after the patient received PRP + MCD, obvious collapse of the subchondral bone of the femoral head could be seen. In our study, patients who received PRP + intersecting femoral head decompression had higher HHS, lower VAS and less progression of ANFH.

Our study is innovative. No previous study on promoting penetration efficiency of cytokines in PRP can be found. Our research offered an effective and innovative solution to PRP treatment of early stage ONFH. Besides PRP, intra-articular injection of MSCs or growth factors may be combined with our method to promote penetration efficiency. The study also has several limitations. Firstly, this is a retrospective design, which has inherent restrictions. A prospective cohort study can be conducted in the future to be more convincing. Secondly, due to patient volume limitations, the sample size may not be sufficient. More patients are needed in the future to prove this conclusion. Thirdly, two years of follow-up may be not enough. Longer follow-up is needed to prove long-term efficacy. Fourthly, MRI is a highly sensitive imaging tool, Due to relatively high prices, MRI was not widely used in our study. Besides, this study lacked histologic evidence. If animal experiments is used, the femoral head can be removed for histologic comparison, which can be more convincing.

## Conclusion

Intersecting femoral head decompression provides a channel for cytokines to enter lesion area of femoral head, promoting osteogenesis and angiogenesis. Compared with the patients who received PRP injection + MCD, patients who received PRP injection + intersecting femoral head decompression had slower progression of ANFH.

## Data Availability

The datasets generated in this investigation are currently restricted from public access due to mandatory ethical compliance requirements regarding patient confidentiality and anonymization standards. However, these data can be obtained by contacting the first author (Lei Sun) with legitimate research purposes.

## Abbreviations

PRP: Platelet-rich plasma
ANFH: Avascular necrosis of femoral head
MCD: Multiple core decompression
HHS: Harris Hip Scores
VAS: Visual analogue scale
